# The Impact of ZnO and Fe_2_O_3_ Nanoparticles on Sunflower Seed Germination, Phenolic Content and Antiglycation Potential

**DOI:** 10.3390/plants13131724

**Published:** 2024-06-21

**Authors:** Waleed Khaled Kaddem Al-Sudani, Rawaa Shakir Shnain Al-Shammari, Mohammed Saheb Abed, Jasim Hafedh Al-Saedi, Maria Mernea, Iulia Ioana Lungu, Florian Dumitrache, Dan Florin Mihailescu

**Affiliations:** 1 Interdisciplinary School of Doctoral Studies, University of Bucharest, 36–46 Mihail Kogălniceanu Bd, 050107 Bucharest, Romania; local.wk@gmail.com; 2 Ministry of Trade in Iraq, The General Company for Foodstuff Trade, Al Mansour, Baghdad 10013, Iraq; 3 Department of Anatomy, Animal Physiology and Biophysics, Faculty of Biology, University of Bucharest, 91–95 Splaiul Independenței Str., 050095 Bucharest, Romania; localra07@gmail.com (R.S.S.A.-S.); jasimali050@gmail.com (J.H.A.-S.); d.f.mihailescu@gmail.com (D.F.M.); 4 Ministry of Agriculture in Iraq, Al Wazeria, Baghdad 10053, Iraq; 5 Doctoral School of Biology, Faculty of Biology, University of Bucharest, 91–95 Splaiul Independenței Str., 050095 Bucharest, Romania; mohammedalglehawy@gmail.com; 6 Al-Mussaib Technical Institute, Al-Furat Al-Awsat Technical University, Babylon 51009, Iraq; 7 National Institute for Laser, Plasma and Radiation Physics, 409 Atomistilor Street, 077125 Magurele, Romania; iulia.lungu@inflpr.ro (I.I.L.); florian.dumitrache@inflpr.ro (F.D.); 8 Biometric Psychiatric Genetics Research Unit, Alexandru Obregia Psychiatric Hospital, Șoseaua Berceni 10 Str., 041914 Bucharest, Romania

**Keywords:** sunflower seeds, nano-priming, ZnO nanoparticles, Fe_2_O_3_ nanoparticles, germination, total phenolics content, protein glycation

## Abstract

The enhancement of seed germination by using nanoparticles (NPs) holds the potential to elicit the synthesis of more desired compounds with important biomedical applications, such as preventing protein glycation, which occurs in diabetes. Here, we used 7 nm and 100 nm ZnO and 4.5 nm and 16.7 nm Fe_2_O_3_ NPs to treat sunflower seeds. We evaluated the effects on germination, total phenolic content, and the anti-glycation potential of extracted polyphenols. Sunflower seeds were allowed to germinate in vitro after soaking in NP solutions of different concentrations. Polyphenols were extracted, dosed, and used in serum albumin glycation experiments. The germination speed of seeds was significantly increased by the 100 nm ZnO NPs and significantly decreased by the 4.5 nm Fe_2_O_3_ NPs. The total phenolic content (TPC) of seeds was influenced by the type of NP, as ZnO NPs enhanced TPC, and the size of the NPs, as smaller NPs led to improved parameters. The polyphenols extracted from seeds inhibited protein glycation, especially those extracted from seeds treated with 7 nm ZnO. The usage of NPs impacted the germination speed and total polyphenol content of sunflower seeds, highlighting the importance of NP type and size in the germination process.

## 1. Introduction

The germination of seeds induces an increase in their concentration of phenolic compounds [1]. Being produced as secondary metabolites, these compounds protect the seeds by having an antioxidant activity [2]. In human health, phenols are recognized for their benefits in conditions like inflammation, diabetes, cardiovascular diseases, cancer or neurodegenerative diseases [3]. Thus, germination is a low-cost, but effective method to improve the nutritional value of seeds [2].

Enhancing the germination of seeds is a subject of interest given the demand to increase the productivity and nutritional value of crops [4,5]. There are different methods to achieve this goal. A pre-sowing treatment of winter wheat seeds with an electromagnetic field stimulated the germination of big seeds that otherwise germinate slower than small seeds [4]. Seed priming with different agents (distilled water, osmotic solutions, phytohormones, cytokinins, microorganisms, chemicals) is also a promising method [6]. For instance, a study conducted on stevia showed that potassium chloride and benzylamine purine lead to improved biochemical attributes of seeds and resulting plants [7]. The priming of seeds also enhances their polyphenol content. Mung beans soaked in CaCl_2_ presented a concentration of polyphenols up to 3.07 times higher than in ungerminated seeds [8]. Also, the extracted polyphenols exhibited an inhibitory effect on α-glucosidase, suggesting a potential hypoglycemic activity in type 2 diabetes [8].

The usage of nanoparticles (NPs) as priming agents (nano-priming) could harness the advantages of NPs like rapid internalization and complex biological effects involving physiological and metabolism modifications [5]. Metal (Fe, Ag, Zn, Mn, Cu), selenium, and metal oxide (ZnO, Fe_3_O_4_) NPs were proven to be efficient priming agents [5,9,10]. Some NPs facilitate electron exchange and enhance surface reaction capabilities with various components present in plant cells and tissues [11]; others can influence the expression of genes that are drought-inducible [12]. Some metal-based NPs can be toxic to seeds due to the re-oxidation process, such as copper oxide NPs [13] or silver NPs [14]. NPs comprising iron, zinc, and manganese have been successfully used in experimental seed nano-priming due to the important role of these metals in plant metabolism and plant biofortification [15,16,17]. In the case of ZnO and Fe_3_O_4_ (magnetite), Sencan et al. [9] showed that their stimulating effect on basil seed germination depends on size, surface area, morphology, and concentration.

In the present work, we addressed the ability of ZnO and γ-Fe_2_O_3_ (hematite) NPs to stimulate the germination of sunflower (*Helianthus annuus*) seeds. Sunflower seeds and sprouts are rich in flavonoids, phenolic acids, trace elements, and vitamins, with important pharmacological activities [18]. We determined the total phenolic content of germinated seeds that were previously soaked in NP solutions. Also, we determined the ability of these polyphenols to inhibit the glycation of proteins, a process specific to diabetes [19]. Polyphenols extracted from different plant parts were shown to inhibit protein glycation [20]. The polyphenols from germinated seeds are no exception. In this respect, phytochemicals from alfalfa sprouts were shown to present a strong inhibition of advanced glycation end product (AGE) formation and enzymes considered to be therapeutic targets in diabetes and obesity [21].

## 2. Results

### 2.1. The Germination of Nano-Primed Seeds

**Figure 1:**
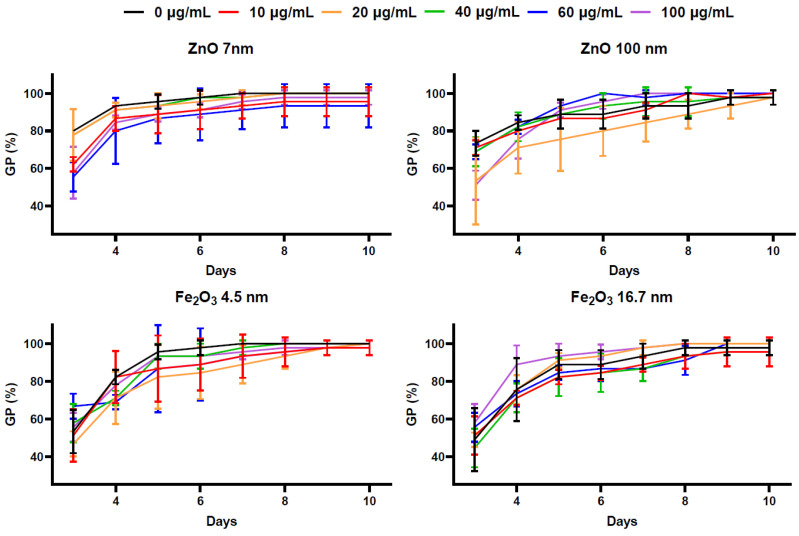
Time course of cumulative germination percentages (GPs) of *Helianthus annuus* seeds treated with different concentrations of NPs, mean ± SD (n = 3).

The germinated seeds from the triplicate batches that were treated with different NPs in various concentrations were counted daily over a period of 10 days. Germination started on the third day for all batches. The seeds reached a high final germination percentage (FGP), as presented in Figure 1. On the 10th day, the smallest germination percentage (GP) was recorded in the case of seeds treated with 60 µg/mL of 7 nm ZnO NPs (93.33 ± 11.54). The other groups presented larger GPs and even 100% germination. The GPs were subjected to arcsin transformation [22,23] and the FGPs were compared. There were no significant differences in the FGPs determined for nano-primed seeds relative to the control (Figure 1), as *p*-values obtained from ANOVA tests exceeded the threshold of 0.05. These results show that the FGPs of seeds were not influenced by the treatment with NPs, regardless of type, size, or concentration.

**Figure 2:**
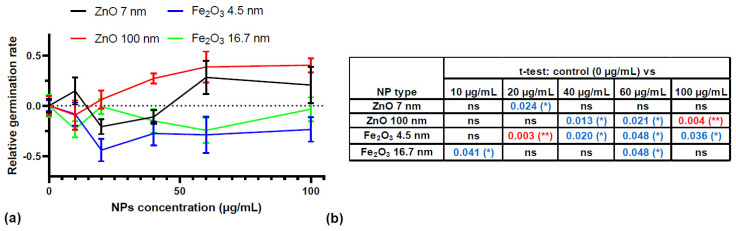
(**a**) Relative germination rates (rate_sample_ − rate_control_) of seeds treated with different concentrations of NPs, mean ± SE (n = 3). (**b**) The *p*-values obtained for pairwise *t*-tests applied to the results from the control of each group (0 µg/mL NPs) vs. results from the treatment with different concentrations of NPs. * *p*-values < 0.05 (highlighted with blue) and ** *p*-values < 0.01 (highlighted with red).

The transformed data were fitted with Gompertz models. Gompertz models are sigmoid models suited to analyzing germination data, revealing information on the kinetics of the process, with superior results in comparison to the logistic regression [24]. Gompertz models fitted the transformed data well, with a median R^2^ value of 0.95. The relative germination rates (calculated as the difference between the germination rate of the samples and the germination rate of the control) obtained for the treatment with the various concentrations of NPs are shown in Figure 2a. The germination rates give information on the speed of the germination process, with higher germination rates being indicative of faster germination. In Figure 2a, the germination rates of the controls were considered as a reference. Negative relative germination rates indicated slower germination than that of the control and positive values indicated faster germination than that of control.

Initially, we addressed the impact of NP concentration on the germination of seeds. Pairwise t-tests performed on the results of treatments with different concentrations of the same NPs relative to the control showed the following: (i) the germination speed of seeds was significantly reduced by 20 µg/mL of 7 nm ZnO NPs, while it did not present significant variations due to the treatment with the other concentrations of 7 nm ZnO NPs; (ii) the treatment with 40, 60, and 100 µg/mL of 100 nm ZnO NPs induced a significant increase in the germination speed of seeds relative to the control; (iii) the treatment with 20, 40, 60, and 100 µg/mL of 4.5 nm Fe_2_O_3_ NPs significantly decreased the germination speed relative to the control; (iv) the only significant differences in the germination speed of seeds induced by the treatment with 16.7 nm Fe_2_O_3_ NPs was seen in the batches treated with 10 and 60 µg/mL NPs, where the germination speed decreased relative to the control.

For the treatment with 100 nm ZnO NPs, 10 or 20 µg/mL of NPs did not produce significant changes in the germination rates of seeds relative to the control or between them. The following dose, 40 µg/mL of NPs, significantly increased the germination speed relative to 20 µg/mL of NPs (t-test *p*-value = 0.03). Enhancing the concentration of NPs used did not induce additional significant changes in the germination rates of seeds relative to 40 µg/mL of NPs. In the case of 4.5 nm Fe_2_O_3_ NPs, the lowest germination rate was seen for the treatment with 20 µg/mL of NPs. Enhancing the concentration of NPs did not induce significant changes in germination rates from one concentration to another.

The treatments with different types of NPs led to significantly different results, as ANOVA comparisons of these data series led to an F statistic of 6.131 and a *p*-value of 0.0039. Post-hoc Tukey tests performed on the data showed significant differences between the results of the treatment with 100 nm ZnO NPs and those of the treatments with 4.5 nm Fe_2_O_3_ NPs (*p*-value = 0.0040) and 16.7 nm Fe_2_O_3_ NPs (*p*-value = 0.0446). This suggests that ZnO NPs enhance the speed of germination, in particular those of 100 nm. The Fe_2_O_3_ NPs decreased germination, in particular those of 4.5 nm. It appears that significant differences in the germination rates were obtained for NPs of extreme sizes—100 nm and 4.5 nm—while the other sizes—7 nm and 16.7 nm—did not induce significant changes relative to the controls.

### 2.2. Total Phenolic Content (TPC) of Seeds Treated with NPs

**Figure 3:**
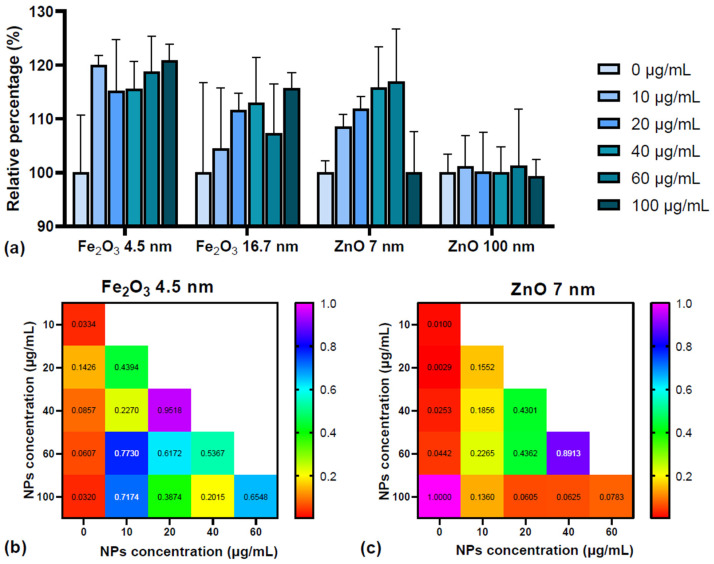
(**a**) TPC percentage in samples relative to the controls (relative percentage = 100 × TPC_sample_/TPC_control_) for the seeds treated with the four types of NPs: 4.5 nm Fe_2_O_3_, 16.7 nm Fe_2_O_3_, 7 nm ZnO, and 100 nm ZnO, means ± SD (n = 3). (**b**,**c**) *p*-values from pairwise t-tests applied to relative percentages of TPC in samples extracted from seeds treated with different concentrations of 4.5 nm Fe_2_O_3_ NPs (**b**) and 7 nm ZnO NPs (**c**). The *p*-values are labeled on the figure and statistically significant *p*-values (<0.05) are colored in red.

The TPC determined for seeds treated with the four types of nanoparticles were used to calculate the relative percentages of the TPC increase or decrease in samples relative to the controls (Figure 3a).

The TPCs for control samples were 0.17 ± 0.03 mg GAE/mL for 16.7 nm Fe_2_O_3_ NPs, 0.17 ± 0.02 mg GAE/mL for 4.5 nm Fe_2_O_3_ NPs, 0.20 ± 0.01 mg GAE/mL for 100 nm ZnO NPs, and 0.19 ± 0.004 mg GAE/mL for 7 nm ZnO NPs. The variation of TPC with the concentration of NPs used in the seed treatments was statistically significant in the case of smaller NPs—4.5 nm Fe_2_O_3_ (ANOVA parameter F = 6.770, *p* = 0.0289) and 7 nm ZnO (ANOVA parameter F = 4.419, *p* = 0.0163)—and not significant in the case of larger nanoparticles—16.7 nm Fe_2_O_3_ (ANOVA parameter F = 1.055, *p* = 0.4310) and 100 nm ZnO (ANOVA parameter F = 0.0448, *p* = 0.9985).

TPC was higher in the samples treated with the small Fe_2_O_3_ and ZnO NPs. The 4.5 nm Fe_2_O_3_ NPs significantly increased TPC content relative to the control in 10 µg/mL and 100 µg/mL doses (Figure 3b). The 7 nm ZnO appeared to induce an increase in the TPC with NP dose, except for the 100 µg/mL NP concentration, where TPC dropped to a value similar to that of the control (Figure 3c). The mean TPC values of seeds treated with 16.7 nm Fe_2_O_3_ NPs were higher than those of the control, but, considering the standard deviations too, the increase was not significant. In the case of seeds treated with 100 nm ZnO NPs, the TPCs of samples were close to control values, regardless of NP concentration. These results show that TPC is influenced by NP size and type; therefore, we detail these aspects in the next section.

#### 2.2.1. Effect of NP Type and Size on TPC

The average TPCs in the datasets of seeds treated with NPs were (i) 0.1873 ± 0.0171 mg/mL for 16.7 nm Fe_2_O_3_ NPs, (ii) 0.1898 ± 0.0154 mg/mL for 4.5 nm Fe_2_O_3_ NPs, (iii) 0.199 ± 0.0106 mg/mL for 100 nm ZnO NPs, and (iv) 0.2083 ± 0.0167 mg/mL for 7 nm ZnO NPs. Since the dataset comprising the results for the TPC content in seeds did not meet the normality assumption (see Supplementary Materials), a Kruskal–Wallis test was applied to determine the differences due to NP type—the test statistic being 13.59 and the *p*-value being 0.00023—and NP size—the test statistic being 15.73 and the *p*-value being 0.00129. The two tests indicated that NP type and size significantly influenced the TPC in the samples.

**Figure 4:**
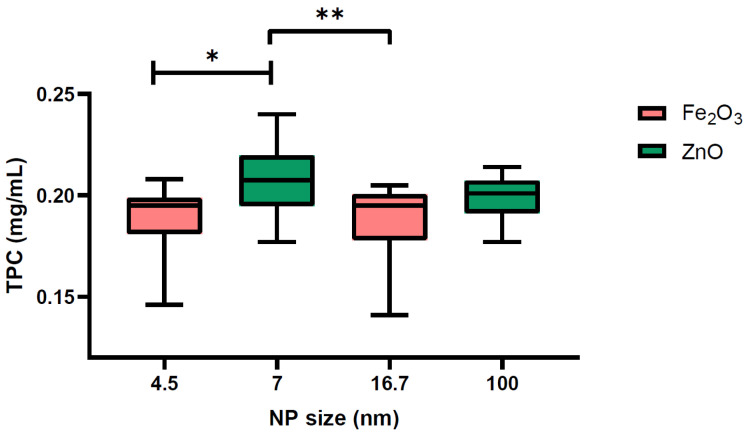
Boxplot representation of the phenolic concentrations in mg/mL associated with the four NP sizes. The statistical significances derived from the Mann–Whitney U test with Bonferroni correction are marked with * for 0.01 < *p* < 0.05 or ** for 0.001 < *p* < 0.01.

To determine the source of difference in the TPCs of seeds treated with different NPs (chemical composition and size), a Mann–Whitney U test with Bonferroni correction was applied. The results for testing the impact of NP type (Fe_2_O_3_ vs. ZnO) were as follows: test statistic = 321, adjusted *p*-value = 0.00023. This is evidence that, indeed, the two types of NPs induced differences in TPC. The impact of NP size on TPC is given in Table 1 and Figure 4. There were statistical differences between the NP sizes of 4.5 nm vs. 7 nm and 7 nm vs. 16.7 nm. Given the impact of both NP type and size on the determined TPCs, we performed an analysis of covariance (ANCOVA) on the data.

#### 2.2.2. TPC Model Based on NP Type and Concentration

ANCOVA was used to compare the results of the treatments while controlling the variability of other quantitative variants (covariates). The preconditions were checked prior to applying the test. The linearity precondition was not fully satisfied as there was only a weak, non-statistically significant linear relationship between NP concentration (covariate) and TPC (dependent variable) (Pearson correlation coefficient = 0.19, *p* = 0.11). The homogeneity of regression slopes precondition was met, with the model including the interaction term between the covariate and the independent variable being statistically significant. Still, the interaction term presented a *p*-value of 0.034, which could suggest an assumption violation. The homoscedasticity (homogeneity of variances) was met, but the evidence was not very strong, with the *p*-value of the Breusch–Pagan test (0.075) being close to 0.05. The normality of residuals precondition was met, with the Shapiro–Wilk test *p*-value being 0.946. The model did not involve multicollinearity and the variance inflation factor (VIF) for both the covariate and the independent variable was ~1.39, below the common threshold (5 or 10). Assumption checking showed that key ANCOVA preconditions were met, but there could have been potential issues from linearity and the homogeneity of regression slopes.

The ANCOVA model was obtained considering the TPC as a dependent variable, NP concentration as the covariate, and NP type as the factor. The model’s R^2^ was 0.284. The ANCOVA model was specified as follows:

TPC = β_0_ + β_1_ × NP conc + β_2_ × NP type + β_3_ × (NP conc × NP type) + ε (1)

where “NP conc” is NP concentration, “NP type” is the type of NP used in the treatment, and “ε” is the error term.

The coefficient of NP concentration was 0.0002 (*p* = 0.006), suggesting that there was a statistically significant, positive relationship between NP concentration and TPC when not considering NP type. The coefficient for NP type was, on average, 0.0236 units higher for ZnO relative to Fe_2_O_3_ (*p* < 0.001), suggesting that the ZnO treatment led to higher TPC values. The interaction term (NP conc x NP type) was −0.0002 (*p* = 0.034). This suggested that the relationship between NP concentration and TPC was different in the case of Fe_2_O_3_ and ZnO NPs.

### 2.3. Inhibition of BSA Glycation by Seed Extracts

**Figure 5:**
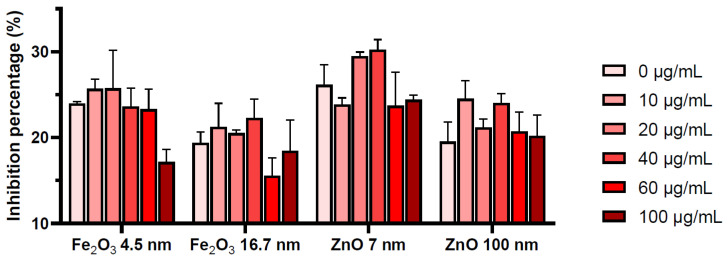
Inhibition percentages of BSA glycation by glucose determined for the polyphenols extracted from seeds treated with increasing amounts of Fe_2_O_3_ and ZnO NPs, means ± SD (n = 3).

BSA was thermally glycated by glucose while untreated or treated with the seed extracts comprising polyphenols. The calculated inhibition percentages are plotted in Figure 5. It can be seen that all extracts inhibited BSA glycation, with the lowest inhibition percentage of 15.53 ± 2.06 determined for the polyphenols extracted from seeds treated with 60 µg/mL of 16.7 nm Fe_2_O_3_ NPs and the highest inhibition percentage of 30.25 ± 1.16 observed in the case of polyphenols extracted from seeds treated with 7 nm ZnO NPs. It also appears that the inhibition percentages were influenced by NP type and size. There also appeared to be differences in the effects of polyphenols extracted after seed treatment with different concentrations of NPs, but their significance needed to be established by applying relevant statistical tests.

Further analysis involved calculating the differences in fluorescence intensity between the positive control (BSA + glucose) and the samples (BSA + glucose + seeds extracts), calculated as ΔF_sample_ = F_control_ − F_sample_. The higher these values, the larger the inhibition percentage. These values were used for applying statistical tests. Precondition checking showed that the ANOVA application raised concerns. The normal distribution of the residuals assessed by the Shapiro–Wilk test showed no significant deviation from normality for data associated with 16.7 nm Fe_2_O_3_ NPs and 7 nm and 100 nm ZnO NPs (*p*-values > 0.05). In the case of results for 4.5 nm Fe_2_O_3_ NPs, a deviation from normality was observed at a 5% significance level (*p* = 0.034). Although the homogeneity of variances precondition was met (Levene’s test statistic = 0.189, *p* = 0.903), the deviation from normality obtained in the case of 4.5 nm Fe_2_O_3_ NPs suggested the usage of non-parametric tests and to apply the ANOVA test with caution.

The non-parametric Kruskal–Wallis H test was applied to compare the average ΔFs across different NP types and sizes. With a test statistic of ~11.35 and a *p*-value of 0.01, it was evident that the obtained differences in the average ΔFs of glycated BSA were significant at the 5% level. In order to determine the specific datasets that differed from the others, a post-hoc analysis was performed by pairwise comparisons through Mann–Whitney U tests with Bonferroni corrections. The analysis pointed to the 16.7 nm Fe_2_O_3_ NPs and 7 nm ZnO NPs as being the only pair presenting significant differences (adjusted *p* = 0.013) in the fluorescence intensity of glycated BSA. This pair of datasets was further analyzed to evaluate the effect size with the significant differences observed between groups, without considering the nanoparticle size as a factor. Cliff’s Delta test gave a value of −1, suggesting that all observations in one group were lower than all observations in the other group. This supports the importance of NP type for the obtained glycated BSA fluorescence intensities and, subsequently, the glycation inhibition percentages.

An ANOVA test was applied to understand the impact of NP type (independent variable) on the results, without considering NP size in the analysis. The one-way ANOVA F-statistic was 4.13 and the *p*-value was 0.031, supporting significant differences at the 5% level for the data resulting from the treatments with different types of NPs. A post-hoc comparison was additionally performed using pairwise t-tests with a Bonferroni correction. This analysis could not identify significant differences between dataset pairs considering the NP types. Although the ANOVA test showed significant differences in the overall effects of the two NP types, the differences are difficult to describe, highlighting the complexity of the results, which may have also been influenced by NP size, NP concentration, and the type of polyphenols occurring in the extracts.

A two-way ANOVA was performed, considering NP type and concentration as independent variables and average ΔFs as the dependent variable. In this context, the main effect of NP type presented an F-statistic of 3.44 and a *p*-value of 0.066. Although this was not statistically significant, the proximity of the *p*-value to the cutoff of 0.05 showed a trend toward the significant impact of NP type on the glycated BSA intensity. The NP concentration was insignificant, with the F-statistic being 1.09 and the *p*-value being 0.391. The interaction between NP type and concentration was also insignificant, with the F-statistic being 0.368 and the *p*-value being 0.866. This shows that there was no significant variation in the effect of NP type over the range of NP concentrations.

The impact of NP type and concentration on glycated BSA fluorescence was also explored by multivariate regression analysis. This model also showed the effect of these variables and their interaction on the average ΔFs. After building the model, the obtained R^2^ value showed that the model explained 45.5% of the variance in the data. The model showed that both NP types (Fe_2_O_3_ and ZnO) presented a tendency to influence the glycated BSA fluorescence, with Fe_2_O_3_ showing a marginally significant effect (*p* = 0.051). The concentration of NPs and the interaction between NP type and concentration were not significant determinants of fluorescence intensity, as their *p*-values exceeded the significance threshold.

## 3. Discussion

The nano-priming of seeds has emerged as a novel and highly effective technique that favorably modifies the metabolism and signaling pathways of seeds [25], thereby impacting various stages of a plant’s lifecycle, such as germination and overall plant growth [26]. Among the multitude of advantages associated with seed nano-priming, the most notable are improved plant growth and development [27], as well as increased nutritional value [28] and increased tolerance to stress [29].

In line with these aspects, here, we investigated the effect of Fe_2_O_3_ and ZnO NPs of different sizes and in different concentrations on the germination of sunflower seeds, their phenolic content, and the value of extracted phenolic compounds in preventing protein glycation. It is interesting to note that both iron and zinc are important nutrients for plants. Iron is an essential co-factor for enzymes, such as cytochrome P450s and Fe(II)/2-oxoglutarate-dependent oxygenase, and is thus an indispensable nutrient in numerous metabolic pathways, including those involved in respiration and photosynthesis [30]. It is known to increase non-enzymatic antioxidant potential when used in seed nano-priming [30]. Zinc, another metal essential for plant metabolism, for which there is estimated to be a 49% global deficiency [31], has also been successfully used in seed nano-priming, especially for combating environmental stress [32].

In our present work, both types of NPs appeared to influence the speed at which the seeds germinated, without influencing the final germination percentage. This is in agreement with other reports in the literature showing that NPs impact the germination of seeds, enhancing or reducing it depending on plant species, NP type, and dosage [33]. A comparison of the effects elicited by the two types of NPs considered here showed that ZnO NPs enhance germination rates, while Fe_2_O_3_ NPs appear to decrease the germination rates of seeds. To our best knowledge, there are not many direct comparative studies between ZnO and Fe_2_O_3_ nanoparticles, especially in the context of seed germination, as the existing literature mostly provides insights into the individual effects of these nanoparticles on plant growth and development [34,35,36]. Our results also showed that larger (100 nm) ZnO NPs are significantly efficient in enhancing germination rates. This result is in agreement with previous studies showing that ZnO NPs enhance the germination of seeds [12,32,37,38].

The results obtained on germination are further supported by the differential impact that NPs have on the TPC levels of germinated seeds. The production of polyphenols in germinating seeds is a defense mechanism against the oxidative stress imposed by the seed’s metabolism and the action of abiotic or biotic stress [39,40]. NPs can induce an additional stress that elicits polyphenol synthesis [9]. In our study, ZnO NPs appeared to significantly increase TPC relative to Fe_2_O_3_ NPs. This result is in agreement with previous studies showing that NP composition can influence bioactivity and impact plant biochemistry [41,42]. In previous studies, ZnO NPs were also reported to improve the germination of seeds of *Capsicum annuum* L. and promote the accumulation of phenolic compounds [43].

Given the antioxidant effects of the phenolic compounds in seeds, we considered testing the efficiency of polyphenols that we extracted from sunflower seeds in inhibiting glycation. The glycation process is favored by reactive oxygen species [44]. Our results confirmed the anti-glycation effect of the extracted polyphenols and revealed its relationship with the NP types and sizes used in the treatment of seeds. Considering the same type of NP, there was no significant impact of NP concentration on the ability of the extracted polyphenols to inhibit BSA glycation. The explanation resides in the glycation inhibition protocol that we used, involving the testing of equal amounts of polyphenols (1 mg/mL of GAE) from all the extracts obtained from the treatments with different concentrations of NPs. The results for TPC content together with the results for glycation inhibition suggest that the different types of NPs trigger the formation of different amounts of polyphenols, with a similar ability to inhibit glycation. While there are many studies highlighting the bioactive properties of plant-derived polyphenols from different sources [20], the literature is sparse on the anti-glycation effects of polyphenols extracted from NP-treated seeds. In this context, we can mention the work of Aloo et al. [21,45], who investigated the antidiabetic effect of compounds extracted from the seeds and sprouts of red cabbage, broccoli, alfalfa, and buckwheat.

Overall, our results showed variability in the TPC and antiglycation effects across the treatments with different NP types and sizes, which supports the distinct nature of nanoparticle–plant interactions. These findings show that the biochemical response of seeds in germination can be tailored using the appropriate NPs to obtain desired health-related outcomes.

## 4. Materials and Methods

### 4.1. Seed Germination

**Figure 6:**
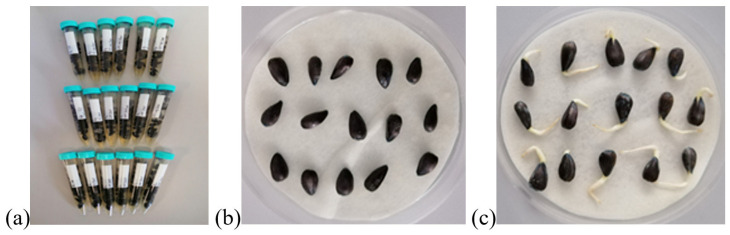
(**a**) Tubes with sunflower seeds soaked in different NP solutions. (**b**) Seeds transferred to a Petri dish immediately after soaking in the NP solution. (**c**) The same seeds after 5 days of germination.

The experiments were performed on seeds of *Helianthus annuus* (sunflower), a Suria hybrid, produced by Ciproma Sem (Bucharest, Romania). A total of 3240 sunflower seeds were prepared by washing with water and disinfection by soaking in 1% sodium hypochlorite for 3 min [46]. NP solutions with concentrations of 10, 20, 40, 60, and 100 µg/mL were prepared using 7 nm ZnO, 100 nm ZnO, 4.5 nm Fe_2_O_3_, and 16.7 nm Fe_2_O_3_ suspended in water. These were used to soak the sunflower seeds for 4 h at 25 °C. The treatments with all NPs in the above-mentioned concentrations, including the condition of 0 µg/mL of NPs representing the control, were applied to 45 seeds in triplicate. After soaking, the seeds were transferred to Petri dishes on moist filter paper and allowed to germinate in the darkness. From each replicate, 30 seeds were removed after 3 days of germination for the extraction of phenolic compounds, as explained in the next section. The remaining 15 seeds in each replicate were allowed to germinate for 10 days (Figure 6). The germinated seeds in each batch were counted and germination percentages (GPs) were calculated by dividing the number of germinated seeds sown in each dish at each time period by the total number of sown seeds, multiplied by 100.

### 4.2. The Extraction and Dosing of Polyphenols

Polyphenols were extracted separately for batches of 10 geminated seeds selected from the 30 seeds that were allowed to germinate for 3 days in each replicate. Germinated seeds were dehulled, weighted, and flaked. These were defatted using 4 mL analytical-grade diethyl ether/1 g of seeds. After the complete removal of the solvent by air-drying, the kernels were mixed with 5 mL ethanol absolute/1 g of kernels. Resulting samples were stored for 24 h at 4 °C. Afterwards, these were centrifuged for 10 min at 14,000 rpm and the supernatant comprising the extracted polyphenols was further analyzed. The total phenolic content (TPC) of the extracts was measured as gallic acid equivalents (GAEs) using the Folin–Ciocalteu method, as previously described in [47]. The calibration curve derived from the gallic acid solutions (concentrations of 0–0.5 mg/mL) was y = 0.156x − 0.035 (R^2^ = 0.007), where y is the solution concentration in mg/mL and x is the absorbance at 765 nm.

### 4.3. The Effect of Extracts on Protein Glycation

The glycation protocol that we applied is described in [47]. In short, 50 mg/mL of BSA was mixed with 0.5 M glucose in 0.1 M phosphate-buffered saline (PBS). The samples were incubated at 50 °C for 4 days. This was the positive control. For the negative control, only BSA was incubated in PBS. In the samples, besides BSA and glucose, we added 1 mg/mL GAE of polyphenols extracted from the germinated seeds. The experiment was performed in triplicate, using the polyphenols that were extracted from each germination replicate. In order to highlight the formation of AGEs, we measured the fluorescence emission of samples considering an excitation wavelength of 335 nm and emission wavelength of 450 nm. The glycation inhibition percentages were calculated as follows:

Inhibition percentage (%) = (1 − F_sample_/F_control_) × 100 (2)

where F_sample_ is the intensity of the fluorescence emission of BSA incubated with glucose and the extracts obtained from seeds treated with 0, 10, 20, 40, 60, and 100 µg/mL of NPs and F_control_ is the intensity of the fluorescence emission of BSA incubated only with glucose.

### 4.4. Chemicals Used in the Study

NPs were obtained from the following sources: (i) 7 nm ZnO was purchased from Alfa Aesar (Ward Hill, MA, USA), (ii) 100 nm ZnO was purchased from Sigma—Aldrich (St. Louis, MO, USA), and (iii) 4.5 nm γ-Fe_2_O_3_ and 16.7 nm γ-Fe_2_O_3_ NPs were synthesized by laser pyrolysis at the National Institute for Laser, Plasma and Radiation Physics, Magurele, Romania [48,49].

Other reagents used in this study were Emsure ^®^ diethyl ether (ACS, ISO, Reag. Ph Eur grade) from Merck (Darmstadt, Germany); ethanol (>99.8% purity) from Sigma-Aldrich (St. Louis, MO, USA); Folin–Ciocalteu’s phenol reagent from Merk (Darmstadt, Germany), BSA (fraction V, >98% purity); and D-(+)-Glucose from Carl Roth (Karlsruhe, Germany).

### 4.5. Data Analysis

The analysis of GPs mentioned in Section 4.1 involved an initial arcsin transformation of data: $y=arcsin(\sqrt{p})$, where p is the proportion of germinated seeds [22]. The transformed data were subjected to variance analysis and were fitted with Gompertz models (Equation (1)) [50] using OriginPro 2015 version b9.2.272. The Gompertz function was as follows:

(3) $$N\left(t\right)=K\cdot e^{{-e}^{-r(t-t_{0})}}$$

where N(t) is the number of germinated seeds at time t, K is the maximum population size (upper asymptote or the maximum number of seeds germinated), r is the germination rate, and t_0_ is the inflection point (time at which the germination rate is the maximum). The germination rates were compared using statistical tests.

The data detailed in Section 4.2 and Section 4.3 were analyzed using the Advanced Data Analysis tool of ChatGPT [51,52]. The conversations with ChatGPT are given in the Supplementary Materials. The tool allowed for the facile application of statistical tests and analyses like the Kruskal–Wallis test [53], Mann–Whitney U test [54] with Bonferroni correction [55], ANOVA (analysis of variance) [56], ANCOVA (analysis of covariance) [57], and multivariate regression analysis.

The application of ANOVA requires the verification of some preconditions: independence of observations, normality of residuals, homogeneity of variances, and balanced design or equal sample size. The normality of residuals was addressed by the Shapiro–Wilk test [58], with the null hypothesis that the residuals were normally distributed. The homogeneity of variances was assessed by Levene’s test [59], with the null hypothesis that there were no significant differences between variances across the groups.

Similarly, the application of ANCOVA can be performed if the following preconditions are met: independence of observations, linearity, homogeneity of regression slopes, homogeneity of variances, normality of residuals, no multicollinearity, reliability of measurement, and additivity. The independence of observations was determined using the Durbin–Watson test [60]. Linearity was addressed by Pearson correlation [61]. The homogeneity of variances (homoscedasticity) was addressed by the Breusch–Pagan test [62]. The normality of residuals was verified by the Shapiro–Wilk test [58]. The multicollinearity was verified by calculating the variance inflation factors (VIFs) [63].

## 5. Conclusions

Here, we investigated the effect of smaller and larger ZnO and Fe_2_O_3_ NPs, applied in increasing concentrations, on sunflower seed germination, TPC, and the anti-glycation potential of extracted phenols. Our results show that NP type and size are the main determinants of their biological effects. The nano-priming of sunflower seeds has no impact on the final germination percentage of seeds, but significantly changes the speed of germination. The larger (100 nm) ZnO NPs led to enhanced germination rates relative to the controls and significantly larger germination rates relative to Fe_2_O_3_ NPs. The latter had decreased germination rates relative to the controls (especially 4.5 nm NPs). Smaller NPs (here, 4.5 nm Fe_2_O_3_ NPs and 7 nm ZnO NPs) led to an increased TPC content. The polyphenols extracted from the seeds that germinated in all conditions possessed the ability to inhibit the glycation of serum albumin. Significant differences between the anti-glycation effects were observed in the case of samples that were treated with 7 nm ZnO or 16.7 nm Fe_2_O_3_ NPs. In this case, the ZnO NPs had a greater effect than Fe_2_O_3_ NPs. Our study brings new evidence of the effect of NPs on seed germination and their polyphenol content, aligning with the current trend of using NPs in agriculture, not just for enhancing crop yield and quality but also for inducing specific biochemical changes that could have health benefits. The antiglycation activity observed underscores the potential of NP-treated plant products as functional foods or ingredients in disease management. Future research should delve deeper into the mechanisms underlying these effects and explore the long-term implications.

## Figures and Tables

**Table 1:** Mann–Whitney U test with Bonferroni correction results on NP size impact on TPC content. The test statistic (TS) and adjusted *p*-value (Adj p) are given for pairs of NP sizes. The insignificant results are marked with NS and the significant results are marked with S and * for 0.01 < *p* < 0.05 or ** for 0.001 < *p* < 0.01.

NP Size	NP Size			
	4.5 nm	7 nm	16.7 nm	100 nm
4.5 nm		TS = 66.5 Adj p = 0.016 (S, *)	TS = 150.5 Adj p = 4.36 (NS)	TS = 105.5 Adj p = 0.456 (NS)
7 nm	TS = 66.5 Adj p = 0.016 (S, *)		TS = 57.5 Adj p = 0.006 (S, **)	TS = 109.5 Adj p = 0.598 (NS)
16.7 nm	TS = 150.5 Adj p = 4.36 (NS)	TS = 57.5 Adj p = 0.006 (S, **)		TS = 91.5 Adj p = 0.16 (NS)
100 nm	TS = 105.5 Adj p = 0.456 (NS)	TS = 109.5 Adj p = 0.598 (NS)	TS = 91.5 Adj p = 0.16 (NS)	

## Data Availability

The data that support the findings of this study are available from the corresponding author upon reasonable request.

## Supplementary Materials

The supporting information can be downloaded at https://www.mdpi.com/article/10.3390/plants13131724/s1.
