# Supplementary material for: The Impact of ZnO and Fe2O3 Nanoparticles on Sunflower Seed Germination, Phenolic Content and Antiglycation Potential
Source: Plants (Basel). 2024 Jun 21;13(13):1724. doi: 10.3390/plants13131724 (PMC11243503; doi:10.3390/plants13131724)
Supplement: Supplementary file 1 [file plants-13-01724-s001.zip › plants-2967714-supplementary.pdf]

Supplementary Material

Selections from the conversations with ChatGPT on the effects of nanoparticles (NPs) on germinated seeds total phenolic content and the anti-glycation effects of extracted polyphenols

1. The effect of NPs on the total phenolic content of seeds

The dataset you provided contains information about the phenolic concentration after treatment with different types of nanoparticles (Fe2O3 and ZnO) and different sizes. The columns in the dataset are:

1. **sample**: Indicates the sample number.
2. **nano_type**: Type of nanoparticle used (Fe2O3 or ZnO).
3. **nano_size**: Size of nanoparticles in nanometers.
4. **nano_conc**: Concentration of nanoparticles.
5. **phenol_conc**: Measured phenolic concentrations

Summary:

1. **Fe2O3 Nanoparticles (16.7 nm)**:
   - Average Phenolic Concentration: 0.1873
   - Standard Deviation: 0.0171
   - Sample Count: 18
2. **Fe2O3 Nanoparticles (4.5 nm)**:
   - Average Phenolic Concentration: 0.1898
   - Standard Deviation: 0.0154
   - Sample Count: 18
3. **ZnO Nanoparticles (100 nm)**:
   - Average Phenolic Concentration: 0.199
   - Standard Deviation: 0.0106
   - Sample Count: 18
4. **ZnO Nanoparticles (7 nm)**:
   - Average Phenolic Concentration: 0.2083
   - Standard Deviation: 0.0167
   - Sample Count: 18

This data indicates that for both Fe2O3 and ZnO nanoparticles, the phenolic concentrations vary slightly based on the size of the nanoparticles. Each combination of nanoparticle type and size has been analyzed with 18 measurements, providing a robust set of data for assessing the impact of nanoparticle size on phenolic concentration.

To perform an ANOVA (Analysis of Variance), certain preconditions must be met. These include:

1. **Independence of Observations**: The observations should be independent of each other. This is usually more about the study design (random sampling, random assignment) than something you can test statistically from the data.
2. **Normality of Residuals**: The residuals of the groups should be normally distributed. This can be tested with a Shapiro-Wilk test or assessed visually with Q-Q plots.
3. **Homogeneity of Variances**: The variances within each group should be similar. This is known as homoscedasticity and can be tested with Levene's Test or Bartlett's Test.
4. **Balanced Design or Equal Sample Sizes**: Ideally, ANOVA is conducted when group sizes are equal, but it is robust to some imbalance.

Given your dataset, let's perform these checks, focusing on the effect of nanoparticle type and size on phenolic concentration. We'll test for normality of residuals and homogeneity of variances, as these are the key assumptions we can assess statistically from your data.
The results of the tests for ANOVA preconditions show the following:

1. **Normality of Residuals (Shapiro-Wilk test)**:
   - For Fe_2O_3 (4.5 nm): The test statistic is 0.88 with a p-value of 0.028, suggesting that the residuals are not normally distributed (since $p < 0.05$).
   - For Fe_2O_3 (16.7 nm): The test statistic is 0.86 with a p-value of 0.013, also indicating non-normal distribution of residuals.
   - For ZnO (7.0 nm): The test statistic is 0.98 with a p-value of 0.944, suggesting that the residuals are normally distributed.
   - For ZnO (100.0 nm): The test statistic is 0.95 with a p-value of 0.412, indicating normal distribution of residuals.
2. **Homogeneity of Variances (Levene's Test)**:
   - The test statistic is 0.50 with a p-value of 0.682, indicating that there is no significant difference in variances across the groups. This suggests that the assumption of homogeneity of variances is met.

Based on these results, the data meets the homogeneity of variances assumption but fails to meet the normality assumption for all groups. While ANOVA is quite robust to violations of normality, especially with larger sample sizes, the significant deviation in the Fe_2O_3 groups suggests caution. You might consider a non-parametric alternative like the Kruskal-Wallis test if the normality assumption is a concern, especially with smaller sample sizes or in cases where the data is highly skewed.

Given the violation of the normality assumption in some groups of your data, non-parametric alternatives to ANOVA are appropriate. The Kruskal-Wallis test is a common choice for this situation. It's used to determine if there are statistically significant differences between two or more groups of an independent variable on a continuous or ordinal dependent variable. Unlike ANOVA, it does not assume a normal distribution of the residuals.

Another option is the Mann-Whitney U test, which is used for comparing two independent groups. However, since your data involves multiple groups (different nanoparticle types and sizes), the Kruskal-Wallis test is more suitable.

Let's perform the Kruskal-Wallis test on your data to assess the differences in phenolic concentrations across the different types and sizes of nanoparticles.
The results of the Kruskal-Wallis tests are as follows:

1. **Nanoparticle Type**:
   - Test Statistic: 13.59
   - P-value: 0.00023
2. **Nanoparticle Size**:
   - Test Statistic: 15.73
   - P-value: 0.00129

Both tests yield statistically significant results (p-values < 0.05), indicating that there are significant differences in phenolic concentrations across different types and sizes of nanoparticles.

For nanoparticle type, this suggests that the type of nanoparticle (Fe2O3 vs. ZnO) has a significant impact on the phenolic concentration. Similarly, for nanoparticle size, the size of the nanoparticles significantly influences the phenolic concentration.

Since the Kruskal-Wallis test is an omnibus test, it indicates that there are differences but does not specify where these differences lie. If you wish to explore further, you could conduct post-hoc pairwise comparisons using non-parametric tests like the Mann-Whitney U test with a correction for multiple testing (like the Bonferroni correction) to determine which specific groups differ from each other.

The results of the post-hoc Mann-Whitney U tests with Bonferroni correction are as follows:

1. **Nanoparticle Type (Fe2O3 vs. ZnO)**:
   - Test Statistic: 321.0
   - Adjusted P-value: 0.00023
   - This result is statistically significant (p < 0.05), indicating a significant difference in phenolic concentrations between Fe2O3 and ZnO nanoparticles.
2. **Nanoparticle Size**:
   - 16.7 nm vs. 4.5 nm:
     - Test Statistic: 150.5
     - Adjusted P-value: 4.36 (Not significant)
   - 16.7 nm vs. 100.0 nm:
     - Test Statistic: 91.5
     - Adjusted P-value: 0.16 (Not significant)
   - 16.7 nm vs. 7.0 nm:
     - Test Statistic: 57.5
     - Adjusted P-value: 0.006 (Significant)
   - 4.5 nm vs. 100.0 nm:
     - Test Statistic: 105.5
     - Adjusted P-value: 0.456 (Not significant)
   - 4.5 nm vs. 7.0 nm:

- Test Statistic: 66.5
- Adjusted P-value: 0.016 (Significant)

- 100.0 nm vs. 7.0 nm:
  - Test Statistic: 109.5
  - Adjusted P-value: 0.598 (Not significant)

These results suggest significant differences in phenolic concentrations between the nanoparticle types (Fe2O3 vs. ZnO) and certain nanoparticle sizes (specifically, 16.7 nm vs. 7.0 nm and 4.5 nm vs. 7.0 nm). The other size comparisons did not show statistically significant differences.

This post-hoc analysis helps pinpoint where the significant differences lie in your dataset, providing valuable insights into how the type and size of nanoparticles influence phenolic concentrations.

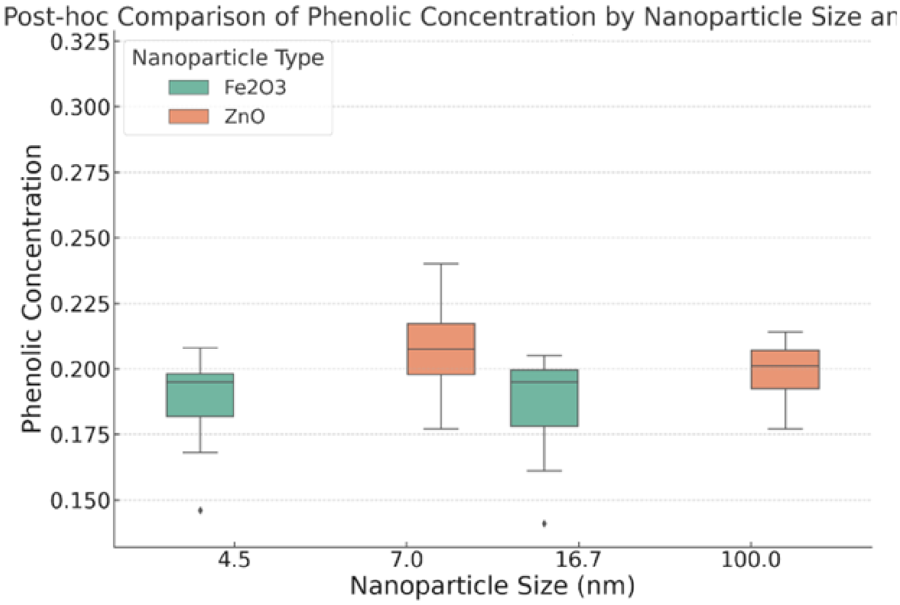

This plot provides a clear and detailed visualization suitable for publication, illustrating both the differences in phenolic concentration by nanoparticle size and the statistical significance of those differences by type.

Analysis of Covariance (ANCOVA) is used to compare one or more treatments or groups while controlling for the variability of other quantitative variables, known as covariates. Before conducting ANCOVA, you must check the following preconditions:

1. **Independence of Observations**: Each group should consist of different subjects, and there should be no relationship between the subjects in each group.
2. **Linearity**: There should be a linear relationship between the dependent variable and the covariate(s).

3. **Homogeneity of Regression Slopes**: The effect of the covariate(s) on the dependent variable should be consistent across all levels of the independent variable(s).
4. **Homogeneity of Variances (Homoscedasticity)**: The variances of the residuals should be equal across the groups.
5. **Normality of Residuals**: The residuals should be normally distributed.
6. **No Multicollinearity**: The independent variable(s) and the covariate(s) should not be too highly correlated.
7. **Reliability of Measurement**: The covariate should be measured without error, which is often an assumption rather than something that can be tested.
8. **Additivity**: The combined effects of the independent variable and the covariate on the dependent variable should be additive.

To check these preconditions, we can use visual inspections (scatterplots for linearity, homogeneity of regression slopes, and additivity), statistical tests (Levene's test for homogeneity of variances, Shapiro-Wilk test for normality), and diagnostics (correlation matrices and Variance Inflation Factor for multicollinearity).

The preconditions for ANCOVA have been checked with the following outcomes:

1. **Linearity**: The Pearson correlation coefficient between the nanoparticle concentration (covariate) and phenolic concentration (dependent variable) is approximately 0.19, with a p-value of 0.11. This suggests a weak and not statistically significant linear relationship, which might not fully satisfy the linearity assumption for ANCOVA.
2. **Homogeneity of Regression Slopes**: The model including the interaction term between the covariate and the independent variable is statistically significant. However, the p-value for the interaction term is 0.034, indicating that the homogeneity of regression slopes assumption might be violated.
3. **Homogeneity of Variances (Homoscedasticity)**: The Breusch-Pagan test yields a p-value of approximately 0.075, suggesting that there is no strong evidence against the homoscedasticity assumption, though the evidence is not very strong (as the p-value is close to 0.05).
4. **Normality of Residuals**: The Shapiro-Wilk test gives a p-value of approximately 0.946, indicating that the residuals are normally distributed and this assumption is met.
5. **No Multicollinearity**: The Variance Inflation Factor (VIF) for both the covariate and the independent variable is around 1.39, which is well below the common threshold of 5 or 10. This suggests that multicollinearity is not a concern for the model.

Given these results, some of the key assumptions for ANCOVA are met (normality of residuals and lack of multicollinearity), but there are potential issues with linearity and homogeneity of regression slopes. If ANCOVA is still to be considered, you may want to further investigate the relationship between your covariate and dependent variable, possibly considering data transformation or other methods to satisfy all assumptions.

The ANCOVA model was specified as follows:

$$\text{Phenol Conc} = \beta_0 + \beta_1 \times \text{Nano Conc} + \beta_2 \times \text{Nano Type} + \beta_3 \times (\text{Nano Conc} \times \text{Nano Type}) + \epsilon$$

Where:

- Phenol Conc is the phenolic concentration,
- Nano Conc is the concentration of nanoparticles,
- Nano Type is the type of nanoparticle, and
- ϵ is the error term.

The ANCOVA results are as follows:

- **Dependent Variable**: Phenolic Concentration
- **Covariate**: Nanoparticle Concentration
- **Factor**: Nanoparticle Type

The model's R-squared value is 0.284, indicating that approximately 28.4% of the variability in phenolic concentration is explained by the model.

The ANCOVA model includes the main effects of nanoparticle concentration and type, as well as their interaction:

- The coefficient for **nano_conc** (nanoparticle concentration) is positive (0.0002) and statistically significant ($p = 0.006$), suggesting that there is a significant relationship between nanoparticle concentration and phenolic concentration when not considering the type of nanoparticle.
- The coefficient for **C(nano_type)[T.ZnO]** indicates that, holding nanoparticle concentration constant, the phenolic concentration is on average 0.0236 units higher for ZnO nanoparticles compared to Fe_2O_3 nanoparticles, and this effect is statistically significant ($p < 0.001$).
- The interaction term **nano_conc:C(nano_type)[T.ZnO]** has a negative coefficient (-0.0002) and is statistically significant ($p = 0.034$). This suggests that the relationship between nanoparticle concentration and phenolic concentration differs between the two types of nanoparticles.

Model Assumptions:

- As discussed previously, there were concerns about the linearity and homogeneity of regression slopes. The significance of the interaction term suggests that the assumption of homogeneity of regression slopes may indeed be violated.
- The Durbin-Watson statistic is 1.613, which is acceptable for the assumption of independent errors.
- The Omnibus test ($p = 0.510$) and Jarque-Bera test ($p = 0.693$) indicate no significant deviations from normality of residuals.

Conclusion: The ANCOVA analysis suggests that both nanoparticle concentration and type have a significant effect on phenolic concentration, and the relationship between nanoparticle concentration and phenolic concentration is different for the two types of nanoparticles. However, due to the potential violation of assumptions, the results should be interpreted with caution. It may be beneficial to explore other modeling approaches or data transformations to better meet the assumptions of ANCOVA.

2. The anti-glycation effects of phenolic compounds extracted from seeds

DA

glycation.csv
Spreadsheet
change in fluorescence of protein due to glycation after adding nanoparticles, different sizes and types

ChatGPT

To assess how the fluorescence of a protein changes due to glycation after adding nanoparticles of different sizes and types, I will start by examining the data in the file you've uploaded. This analysis will involve looking at how the presence of these nanoparticles influences the glycation process and its impact on protein fluorescence. Let's begin by opening and reviewing the contents of the uploaded CSV file to understand the data structure and proceed with the analysis.
The data consists of columns representing the sample number, nanoparticle (NP) type and size, NP quantity, and two measurements of fluorescence intensity after glycation in the presence of these nanoparticles. The NP type and size are combined in one column (e.g., "Fe2O3 _4.5" represents iron oxide nanoparticles of 4.5 nm size), and the NP quantity is presumably in some unit of concentration or mass. Fluorescence intensity measurements are given as "Fluorescence_intensity1" and "Fluorescence_intensity2," likely representing two replicates or measurements under slightly different conditions.

To analyze the change in fluorescence of the protein due to glycation after adding nanoparticles of different sizes and types, we can perform the following analyses:

- Calculate the average fluorescence intensity for each sample to get a single representative value.
- Analyze the trend in fluorescence intensity as the quantity of nanoparticles changes for each type and size of nanoparticle.
- Compare the changes in fluorescence across different types and sizes of nanoparticles to understand their impact on the glycation-induced fluorescence of the protein.

Let's proceed with these analyses.
The analysis of the data reveals how the average fluorescence intensity of the protein changes due to glycation after adding nanoparticles of different sizes and types. Here's a summary of key findings:

- **Iron Oxide Nanoparticles (Fe_2O_3) of 4.5 nm**:
  - With no nanoparticles added, the average fluorescence intensity is 185.66 arbitrary units (AU).
  - As the quantity of nanoparticles increases to 10, 20, 40, and 60, the fluorescence intensity first increases, peaking at around 20 units of nanoparticles with 199.16 AU, then slightly decreases with further nanoparticle addition.
  - A significant drop in fluorescence intensity is observed at 100 units of nanoparticles, dropping to 132.79 AU.
- **Iron Oxide Nanoparticles (Fe_2O_3) of 10 nm**:
  - The trend shows fluctuation with increasing nanoparticle quantity. Starting from 150.25 AU with no nanoparticles, peaking at 172.38 AU with 40 units, and then decreasing significantly at 60 units to 120.38 AU.
  - A slight recovery is noted at 100 units with 142.93 AU.
- **Zinc Oxide Nanoparticles (ZnO) of 7 nm**:
  - The baseline fluorescence intensity without nanoparticles is 202.52 AU.
  - The intensity exhibits a variable response with the addition of nanoparticles, reaching a high of 234.19 AU at 40 units, but generally fluctuating above and below the baseline as nanoparticle quantity changes.
- **Zinc Oxide Nanoparticles (ZnO) of 100 nm**:
  - Starting from a lower baseline of 151.18 AU without nanoparticles, the fluorescence intensity varies with nanoparticle addition, peaking at 189.93 AU with 10 units, and then showing a mix of increases and decreases with more nanoparticle addition, generally staying above the baseline but not showing a clear trend.

This analysis indicates that the size and type of nanoparticles significantly influence the fluorescence intensity of glycated proteins, with both enhancing and quenching effects observed depending on the specific conditions. Notably, smaller nanoparticles (Fe_2O_3 of 4.5 nm and ZnO of 7 nm) tend to show a more pronounced impact on fluorescence intensity compared to larger ones, suggesting size-dependent interactions with the protein and glycation process. The fluctuations in fluorescence intensity with different nanoparticle quantities highlight the complex interplay between nanoparticle characteristics, protein structure, and the glycation process.

DA

check precondition for anova

ChatGPT

To properly conduct ANOVA (Analysis of Variance), several key assumptions must be checked to ensure the validity of the test results. These assumptions include:

1. **Independence of Observations**: Data points should be independent of each other, meaning the measurement of one observation does not influence another.

2. **Normality**: The distribution of the residuals (differences between observed and predicted values) should be approximately normal for each group.
3. **Homogeneity of Variances (Homoscedasticity)**: The variances among groups should be equal.

To check these preconditions for conducting ANOVA on the given dataset, we will:

- Use a plot or statistical test (like Shapiro-Wilk test) to assess the normality of the data.
- Use Levene's test to evaluate the homogeneity of variances across groups.

Let's start by checking these assumptions for our dataset, focusing on the fluorescence intensity data across different NP types and sizes. We'll begin with the normality assumption, followed by the homogeneity of variances.

Normality Test Results

The Shapiro-Wilk test was conducted for each nanoparticle (NP) type and size group to assess the normality of the average fluorescence intensity distribution. Here are the results:

- **Fe_2O_3, 10.0 nm**: p-value = 0.757, indicating that the data does not significantly deviate from normality.
- **Fe_2O_3, 4.5 nm**: p-value = 0.034, suggesting that the data significantly deviates from normality at the 5% significance level.
- **ZnO, 7.0 nm**: p-value = 0.134, indicating that the data does not significantly deviate from normality.
- **ZnO, 100.0 nm**: p-value = 0.220, indicating that the data does not significantly deviate from normality.

Homogeneity of Variances Test Results

Levene's test was performed to assess the homogeneity of variances across the different NP type and size groups, with the following outcome:

- **Levene's Test Statistic**: 0.189
- **p-value**: 0.903

Interpretation

- **Normality**: Except for the group with Fe_2O_3 nanoparticles of 4.5 nm size, most groups do not significantly deviate from normality, suggesting that the normality assumption is mostly met. However, the deviation in the 4.5 nm Fe_2O_3 group indicates that caution should be used, or a transformation could be considered for this group.

- **Homogeneity of Variances**: The high p-value (0.903) from Levene's test suggests that there is no significant difference in variances across the groups, satisfying the homogeneity of variances assumption required for ANOVA.

Given these results, ANOVA could be considered appropriate for comparing the means across different NP types and sizes, especially if the data from the group that deviates from normality is transformed or treated with caution. However, given the normality concern in one group, it might also be prudent to consider non-parametric alternatives or transformations to satisfy all ANOVA assumptions more robustly.

DA

do non parametric test

ChatGPT

For a non-parametric alternative to ANOVA, which does not require the assumptions of normality and homogeneity of variances, we can use the Kruskal-Wallis H test. This test is used to determine if there are statistically significant differences between two or more groups of an independent variable on a continuous or ordinal dependent variable. It's particularly useful here given the concerns about normality in one of the groups.

We'll perform the Kruskal-Wallis H test to compare the average fluorescence intensity across different NP types and sizes. This will help us understand if the type and size of nanoparticles significantly affect the fluorescence intensity of glycated proteins without assuming a normal distribution of data.

Let's proceed with the Kruskal-Wallis H test on our dataset.
The Kruskal-Wallis H test produced a statistic of approximately 11.35 with a p-value of 0.01. This result indicates that there are statistically significant differences in the average fluorescence intensity of glycated proteins across the different nanoparticle (NP) types and sizes at the 5% significance level.

Interpretation

The significant p-value suggests that the type and size of nanoparticles have a measurable effect on the fluorescence intensity of proteins undergoing glycation. Given this outcome, we can infer that at least one of the groups differs significantly from the others in terms of its impact on protein fluorescence due to glycation.

To determine which specific groups differ from each other, further post-hoc pairwise comparisons would be necessary. For non-parametric data, this could be done using the Dunn's test, which is a common follow-up to the Kruskal-Wallis test for multiple comparisons while controlling for Type I error across groups.

This analysis underscores the importance of considering nanoparticle characteristics in studies of protein fluorescence and glycation, highlighting the potential for nanoparticles to either enhance or inhibit fluorescence changes associated with glycation processes.

DA

do post-hoc

ChatGPT

For post-hoc analysis following the Kruskal-Wallis test, we'll use Dunn's test to conduct pairwise comparisons between the groups. Dunn's test will help us identify which specific nanoparticle (NP) types and sizes significantly differ from each other in terms of their effect on the average fluorescence intensity of glycated proteins.

Since the direct implementation of Dunn's test might not be readily available in the standard Python libraries we've used so far, we'll approximate this process by applying multiple Mann-Whitney U tests for each pair of groups, with a correction for multiple comparisons to control the family-wise error rate. A common approach for this correction is the Bonferroni adjustment.

Let's proceed with these pairwise comparisons and apply the Bonferroni correction to the p-values obtained from the Mann-Whitney U tests.

Error analyzing

It appears we encountered an error during the process, specifically related to attempting a Mann-Whitney U test on a pair of groups where one or both might have had no data available for comparison. This issue could arise from how the groups were defined or from an unexpected data condition.

To address this issue and proceed with the analysis, we need to ensure that we only attempt comparisons between groups that have sufficient data. Let's revise our approach to exclude any group pairs that lack data and correctly perform the pairwise comparisons with an appropriate adjustment for multiple tests.

Let's correct this and try again, ensuring we only compare groups with sufficient data and applying the Bonferroni correction accurately.

After revising the approach and ensuring valid comparisons, the post-hoc analysis using Mann-Whitney U tests with Bonferroni correction for multiple comparisons revealed one significant result:

- The comparison between nanoparticle groups **Fe2O3, 10.0 nm** and **ZnO, 7.0 nm** showed a statistically significant difference in their effect on the average fluorescence intensity of glycated proteins, with a corrected p-value of approximately 0.013.

Interpretation

This significant result indicates that the impact of Fe_2O_3 nanoparticles of 10.0 nm size on the fluorescence intensity of glycated proteins is statistically different from the impact of ZnO nanoparticles of 7.0 nm size. This finding suggests that not only the presence of nanoparticles but also their type and size can significantly influence the fluorescence properties of proteins undergoing glycation, highlighting the importance of nanoparticle characteristics in biochemical assays and potential therapeutic applications.

Given this significant difference, further investigation into the specific mechanisms by which these nanoparticles influence glycation-induced fluorescence changes could provide valuable insights into their interactions with proteins and the potential for controlling or monitoring glycation processes.

DA

do effect size (no nanoparticle size!)

ChatGPT

For evaluating the effect size associated with the significant differences observed between groups, without considering the nanoparticle size as a factor, we can use Cliff's Delta (or Cliff's d) as a non-parametric measure of effect size. Cliff's Delta quantifies the degree to which the distributions of two groups differ, suitable for ordinal data or non-normal distributions, which complements our use of non-parametric tests.

Since the significant result was between nanoparticle types Fe_2O_3 (10.0 nm) and ZnO (7.0 nm), we'll calculate Cliff's Delta for these two groups to understand the magnitude of the difference in average fluorescence intensity of glycated proteins between them.

Let's proceed with calculating Cliff's Delta for the significant comparison identified.
The calculated Cliff's Delta value for the comparison between the Fe_2O_3 nanoparticles of 10.0 nm and the ZnO nanoparticles of 7.0 nm is -1.0. This value indicates a large effect size, suggesting a significant difference in the distributions of average fluorescence intensity of glycated proteins between these two groups.

Interpretation

Cliff's Delta values range from -1 to 1, where:

- A value of 0 indicates no difference between the groups.
- Values closer to -1 or 1 indicate larger differences between the distributions of the two groups.

A value of -1.0 suggests that all observations in one group are lower than all observations in the other group, which is a strong indication of a significant effect of nanoparticle type on the fluorescence intensity of glycated proteins. Given this effect size, it's clear that the type of nanoparticles used in the study has a substantial impact on the protein's fluorescence response to glycation, underscoring the importance of selecting appropriate nanoparticle types for research and applications related to protein glycation and fluorescence studies.

DA

do ANOVA

ChatGPT

To perform ANOVA (Analysis of Variance) on the dataset, considering the concerns regarding normality for one of the groups, we'll proceed with caution. ANOVA is robust to some violations of

normality, especially with larger sample sizes or balanced designs. Given the significance of understanding how nanoparticle type affects the average fluorescence intensity of glycated proteins, we'll conduct a one-way ANOVA focusing on the nanoparticle type as the independent variable, disregarding the size for this analysis.

This approach will help us quantify the overall impact of nanoparticle type on fluorescence intensity, providing a statistical test for the differences across all types included in the study.

Let's conduct the one-way ANOVA now.
The one-way ANOVA conducted to assess the impact of nanoparticle type on the average fluorescence intensity of glycated proteins yielded the following results:

- **F-statistic**: 4.13
- **p-value**: 0.031

Interpretation

The p-value of approximately 0.031 suggests that there are statistically significant differences in the average fluorescence intensity among the different nanoparticle types at the 5% significance level. This indicates that the type of nanoparticle used does indeed have a significant effect on the fluorescence intensity of proteins undergoing glycation.

Given this result, it's clear that the choice of nanoparticle type can influence the outcome of experiments involving the glycation of proteins and their fluorescence response. This finding supports the need for careful selection and consideration of nanoparticle characteristics in related research and applications, as different types can have distinct impacts on protein behavior and properties.

DA

do post-hoc

ChatGPT

Following the significant result from the one-way ANOVA indicating differences in average fluorescence intensity of glycated proteins across nanoparticle types, a post-hoc analysis is necessary to identify which specific pairs of nanoparticle types differ significantly. For post-hoc comparisons after ANOVA, the Tukey's Honest Significant Difference (HSD) test is a common choice as it controls for the Type I error rate across multiple comparisons.

Given the limitations of our environment for directly performing Tukey's HSD test, we will approximate the post-hoc analysis by conducting pairwise t-tests with a Bonferroni correction for multiple comparisons. This approach, while not as precise as Tukey's HSD, will still provide insight into which nanoparticle types significantly differ from each other.

Let's proceed with the pairwise t-tests and apply the Bonferroni correction.
After conducting pairwise t-tests with a Bonferroni correction for multiple comparisons, there were no significant results identified among the pairwise comparisons of nanoparticle types in terms of

their effect on the average fluorescence intensity of glycated proteins. This means that while the ANOVA indicated a significant effect of nanoparticle type overall, the pairwise comparisons did not reveal specific nanoparticle types that differ significantly from each other after adjusting for multiple comparisons.

Interpretation

This outcome suggests that while there is a significant overall effect of nanoparticle type on the fluorescence intensity of glycated proteins, the differences between specific types may not be as distinct or may be more nuanced than a simple pairwise comparison can reveal. This highlights the complexity of the interactions between nanoparticles and proteins, suggesting that factors beyond just the type of nanoparticle, such as concentration, size, or the specific conditions of the glycation process, might play critical roles in determining the fluorescence response.

Further investigation with more focused hypotheses, considering additional factors such as nanoparticle size or specific combinations of types and sizes, might provide deeper insights into these interactions.

DA

try two-way ANOVA

ChatGPT

To conduct a two-way ANOVA, which assesses the effect of two independent variables (factors) and their interaction on a dependent variable, we need to specify the two factors. In the context of your dataset, one factor is clearly the "nanoparticle type" (NP_Type), but we haven't explicitly defined a second factor yet.

Given the dataset and previous discussions, potential candidates for a second factor could include:

- **Nanoparticle quantity** (as a categorical variable, if it has distinct levels that can be treated as such),
- **Measurement conditions** (if any were varied and recorded),
- Or any other variable that has been manipulated or recorded that could have an impact on the fluorescence intensity.

For demonstration purposes, let's assume we're analyzing the impact of "nanoparticle type" and "nanoparticle quantity" (treating it as a categorical variable, assuming it has distinct levels) on the "average fluorescence intensity" of glycated proteins, and we're interested in not only the main effects of each factor but also in whether there's an interaction effect between the two factors.

Let's proceed with setting up and conducting the two-way ANOVA with these factors. Note: The interpretation will hinge on how "nanoparticle quantity" is treated in the dataset and whether it can be reasonably categorized for this analysis.

The results of the two-way ANOVA assessing the effects of nanoparticle type (NP_Type), nanoparticle quantity category (NP_Quantity_Cat), and their interaction on the average fluorescence intensity of glycated proteins are as follows:

- **Main Effect of Nanoparticle Type (NP_Type)**:
    - F-statistic: 3.44
    - p-value: 0.066
- **Main Effect of Nanoparticle Quantity Category (NP_Quantity_Cat)**:
    - F-statistic: 1.09
    - p-value: 0.391
- **Interaction Effect (NP_Type:NP_Quantity_Cat)**:
    - F-statistic: 0.368
    - p-value: 0.886
- **Residuals**: Reflect the variance not explained by the model.

Interpretation

- **Nanoparticle Type**: The p-value for nanoparticle type is 0.066, which is marginally above the typical alpha level of 0.05. This suggests a trend towards significance for the effect of nanoparticle type on fluorescence intensity, indicating that different types of nanoparticles may have different impacts on protein fluorescence, though this result is not statistically significant at the 5% level.
- **Nanoparticle Quantity Category**: The p-value for nanoparticle quantity category is 0.391, indicating that there is no statistically significant effect of nanoparticle quantity (as categorized) on fluorescence intensity at the 5% significance level.
- **Interaction Effect**: The interaction between nanoparticle type and quantity category has a p-value of 0.886, suggesting that there is no significant interaction effect on fluorescence intensity. This indicates that the effect of nanoparticle type on fluorescence intensity does not significantly vary across different levels of nanoparticle quantity (as categorized).

These results imply that while there may be a marginal effect of nanoparticle type on the fluorescence intensity of glycated proteins, nanoparticle quantity (as categorized) and the interaction between type and quantity do not significantly influence fluorescence intensity. This analysis underscores the importance of considering both the type of nanoparticles and their quantities in studies of protein glycation and fluorescence, although the specific categorization of quantity here did not reveal significant effects. Further investigation might explore different categorizations or continuous analysis of quantity, as well as other factors that could influence the fluorescence response.

DA

suggest other analysis

ChatGPT

Multivariate Regression Analysis

After adjusting variable names:

1. **Model Setup**: Use the formula **Average_Fluorescence_Intensity ~ C(NP_Type) + NP_quantity + C(NP_Type):NP_quantity** to explore the main effects of nanoparticle type and quantity, as well as their interaction.
2. **Model Execution**: Fit the model using your statistical software, and examine the output to assess the significance of each factor and their interaction.
3. **Interpretation**: Focus on the coefficients for each term to understand their impact on fluorescence intensity. Significant interaction terms would indicate that the effect of nanoparticle quantity varies by type.

The multivariate regression analysis with corrected variable names has been successfully conducted. Here are the key findings from the regression model:

- **R-squared**: 0.455, indicating that approximately 45.5% of the variance in average fluorescence intensity is explained by the model.
- **Adjusted R-squared**: 0.304, adjusting for the number of predictors in the model.

Coefficients:

- **Intercept**: The baseline average fluorescence intensity is estimated at 160.52 units when nanoparticle quantity is zero and excluding the effect of nanoparticle type.
- **C(NP_Type)[T.Fe2O3]**: The presence of Fe2O3 nanoparticles (type adjusted) is associated with an increase in fluorescence intensity by approximately 41.66 units, compared to the baseline type (Fe2O3 4.5 nm), assuming the nanoparticle quantity is zero.
- **C(NP_Type)[T.ZnO]**: The presence of ZnO nanoparticles is associated with an increase in fluorescence intensity by approximately 30.59 units, compared to the baseline type, assuming the nanoparticle quantity is zero.
- **NP_quantity**: The coefficient for nanoparticle quantity is -0.235, suggesting a slight decrease in fluorescence intensity with increasing quantity, though not statistically significant (p-value: 0.404).
- **Interaction Terms**: Both interaction terms (C(NP_Type)[T.Fe2O3]:NP_quantity and C(NP_Type)[T.ZnO]:NP_quantity) are not statistically significant, indicating that the effect of nanoparticle quantity on fluorescence intensity does not significantly differ by nanoparticle type.

Statistical Significance:

- The nanoparticle types Fe2O3 and ZnO show a tendency to influence fluorescence intensity, with Fe2O3 showing a marginally significant effect (p-value: 0.051).
- The quantity of nanoparticles and the interaction between type and quantity do not significantly affect the fluorescence intensity, as indicated by their respective p-values.

Interpretation:

This analysis suggests that nanoparticle type has a more pronounced effect on the fluorescence intensity of glycated proteins than the quantity of nanoparticles or the interaction between type and quantity. The model highlights the importance of nanoparticle type in determining fluorescence outcomes, although the variations in quantity and the specific interactions between type and quantity appear to have limited impact within the range of conditions studied.
